# ﻿Assessing the effect of local heterogeneity on anuran diversity in the Serra da Capivara National Park, Piauí State, Brazil

**DOI:** 10.3897/zookeys.1236.138858

**Published:** 2025-05-05

**Authors:** Kássio de Castro Araújo, Nayla Letícia Rodrigues Assunção, Mirco Solé, Etielle Barroso de Andrade

**Affiliations:** 1 Grupo de Pesquisa em Biodiversidade e Biotecnologia do Centro-Norte Piauiense, Instituto Federal de Educação, Ciência e Tecnologia do Piauí, Campus Pedro II, 64255-000, Pedro II, Piauí, Brasil Instituto Federal de Educação, Ciência e Tecnologia do Piauí Pedro II Brazil; 2 Programa de Pós-graduação em Ecologia e Conservação da Biodiversidade, Departamento de Ciências Biológicas, Universidade Estadual de Santa Cruz, Rodovia Jorge Amado km 16, 45662-900, Ilhéus, Bahia, Brasil Universidade Estadual de Santa Cruz Ilhéus Brazil; 3 Departamento de Ciências Biológicas, Universidade Estadual de Santa Cruz, Rodovia Ilhéus-Itabuna, km 16, 45662-900, Ilhéus, Bahia, Brasil Museum Koenig Bonn (ZFMK), Leibniz Institute for the Analysis of Biodiversity Change Bonn Germany; 4 Museum Koenig Bonn (ZFMK), Leibniz Institute for the Analysis of Biodiversity Change, Adenauerallee 160, 53113 Bonn, North Rhine-Westphalia, Germany Instituto Federal de Educação Ciência e Tecnologia do Piauí Pedro II Brazil

**Keywords:** Abundance, amphibians, biodiversity patterns, Caatinga, checklist, conservation unit, semiarid, species richness

## Abstract

Anurans are among the most diverse groups of vertebrates globally, and environmental heterogeneity plays a key role in shaping their diversity patterns. This study aimed to update the anuran checklist of the Serra da Capivara National Park, Piauí State, northeastern Brazil, and investigate the influence of local heterogeneity on anuran abundance and richness. We recorded 16 anuran species across five families – Bufonidae, Hylidae, Leptodactylidae, Microhylidae, and Phyllomedusidae – most of which are typical Caatinga species or widely distributed taxa. Our results indicate that local heterogeneity did not significantly affect species richness; however, it had a notable impact on anuran abundance. We highlight the importance of heterogeneous habitats in supporting larger anuran populations and enhancing population stability. This study contributes to the understanding of biodiversity patterns and the primary environmental factors affecting anuran communities in Serra da Capivara National Park, offering insights to inform current and future conservation strategies.

## ﻿Introduction

Amphibians are among the most diverse vertebrate groups, with 8827 species registered worldwide (Frost 2024). They were the first vertebrates to colonize terrestrial environments in the Devonian period, approximately 300 million years ago (Bray and Lawson 1985). Morphophysiological adaptations enabled amphibians to occupy a wide range of habitats, including aquatic, terrestrial, and arboreal environments (Vitt and Caldwell 2013), and their varied reproductive strategies (Crump 2015) have contributed significantly to the group’s diversification. Most amphibian species are found in Neotropical regions (Duellman and Trueb 1994), with Brazil hosting the highest amphibian diversity worldwide, where anurans dominate in terms of species richness (Segalla et al. 2021).

Anurans play vital ecological roles, participating in various trophic interactions (Toledo et al. 2007; Ceron et al. 2019) and serving as bioindicators of environmental quality (Lebboroni et al. 2006; Calderon et al. 2019). As such, they contribute to maintaining ecosystem stability and functions (Hocking and Babbitt 2014). Anurans are present in all Brazilian biomes, including the semiarid Caatinga biome (Garda et al. 2017) in northeastern Brazil, which, despite being historically understudied, harbors a high diversity of species. To date, 116 anuran species have been cataloged in the Caatinga, including several endemic taxa (Silva 2022).

Among the states within the Caatinga biome, Piauí is one of the few to have an anuran checklist with 54 species registered (Roberto et al. 2013). The last decade has seen a surge in herpetofaunal research in the state, primarily conducted within Conservation Units (UCs) such as National Parks (e.g., Dal-Vechio et al. 2016; Araújo et al. 2020a; Marques et al. 2023) and Environmental Protection Areas (e.g., Andrade et al. 2016; Araújo et al. 2020b). Nevertheless, unprotected areas outside these UCs also harbor a rich anuran fauna (Benício et al. 2014, 2015). Of the 44 UCs in Piauí, herpetological studies have been conducted in only eight (Pantoja et al. 2022), and some of these are still considered under-sampled, as is the case with Serra da Capivara National Park, where only seven anuran species have been documented so far (Calvacanti et al. 2014).

Understanding the main drivers of anuran diversity is a complex task, as these animals are highly sensitive to environmental conditions (Hopkins 2007). Local habitat heterogeneity has been identified as a key factor influencing anuran diversity in otherwise homogeneous landscapes (e.g., Afonso and Eterovick 2007; Silva et al. 2011; Andrade et al. 2019; Mausberg et al. 2023), including in Piauí State (Andrade et al. 2016; Araújo et al. 2018). According to this hypothesis, more heterogeneous areas tend to support greater species diversity (MacArthur and MacArthur 1961). However, it remains unclear which specific environmental variables play the most significant role in shaping anuran diversity patterns. To address this knowledge gap, we (i) characterized the anuran fauna of Serra da Capivara National Park (SCNP), Piauí State, Brazil, and (ii) tested how local habitat heterogeneity influences anuran abundance and species richness.

## ﻿Material and methods

### ﻿Study area

**Figure 1.**
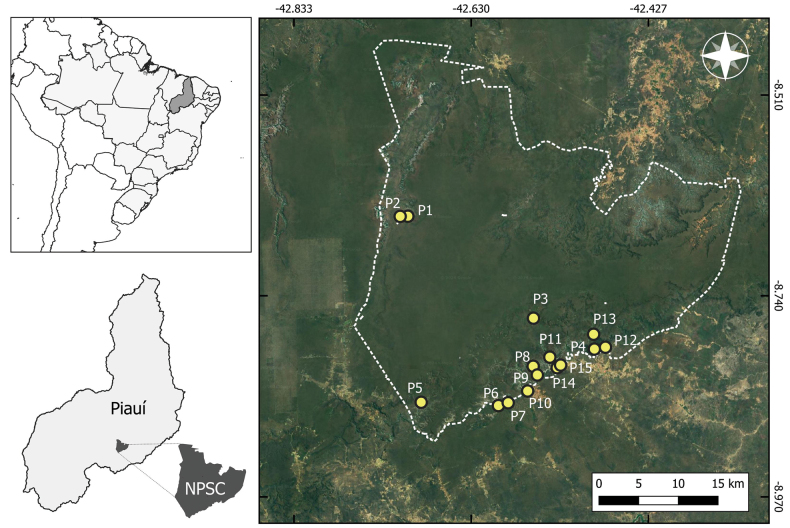
Geographical location of the Serra da Capivara National Park (SCNP), Piauí State, northeastern Brazil, with the distribution of the 15 sampling points.

This study was conducted in the Serra da Capivara National Park (SCNP), located in the state of Piauí, northeastern Brazil (Fig. 1). The park, established by Federal Decree No. 83.548 on June 5, 1979, spans a total area of 130,000 hectares (BRASIL 1990). Although SCNP is situated within the Caatinga biome (IBGE 2019), it is characterized by a mosaic of vegetation types with high species richness. The area includes a variety of vegetation forms, such as tall and dense shrublands, arboreal communities, medium-density woodlands, low shrublands, and mixed shrub-arboreal habitats (Lemos 2004). Rainfall is concentrated primarily between November and April, with an average annual precipitation exceeding 600 mm and a mean temperature of 26 °C (Lemos and Rodal 2002; Aquino and Oliveira 2017).

### ﻿Sampling

We conducted four expeditions, each lasting five consecutive days, from December 2023 to April 2024 in SNCP, totaling 20 sampling days. We used visual and auditory searches (Heyer et al. 1994) in different environments used by anurans within the park. A total of 15 sampling points were randomly chosen according to the vocalization activities of the anurans (Table 1). Fieldwork was carried out by five researchers, beginning at 18:00 h and concluding at 00:00 h each night. We surveyed three points per night, spending approximately 1.5 hours at each site. This resulted in a total sampling effort of 600 hours (5 researchers × 120 hours). A single voucher specimen for each species was collected and deposited in the Coleção Biológica of the Instituto Federal de Ciência e Tecnologia do Piauí, Campus Pedro II (CBPII), Piauí State, northeastern Brazil.

**Table 1.** Environmental description of the 15 sampling points in the Serra da Capivara National Park, Piauí State, northeastern Brazil.

Sampling points	Geographic coordinates	Description
P1	8°38.92'S, 42°42.15'W	Artificial drinking fountain surrounded by shrubs and trees
P2	8°38.93'S, 42°42.68'W	Artificial drinking fountain surrounded by shrubs and trees
P3	8°45.97'S, 42°33.52'W	Temporary pond with shrub and tree vegetation inside and on the edge of the pond
P4	8°48.07'S, 42°36.55'W	Temporary pond with shrub and tree vegetation inside and on the edge of the pond
P5	8°51.72'S, 42°41.24'W	Rocky outcrop modified to accumulate water for longer, presence of thorny shrub and tree vegetation
P6	8°51.94'S, 42°35.93'W	Artificial drinking fountain surrounded by shrubs and trees
P7	8°51.75'S, 42°35.26'W	Modified passage that accumulates water surrounded by shrubs and trees
P8	8°49.24'S, 42°33.54'W	Modified pond located inside the cave
P9	8°49.85'S, 42°33.25'W	Modified passage that accumulates water surrounded by shrubs and trees
P10	8°50.94'S, 42°33.92'W	Modified pond located inside the cave
P11	8°48.62'S, 42°32.40'W	Artificial drinking fountain surrounded by shrubs and trees
P12	8°47.94'S, 42°28.57'W	Temporary pond with shrub and tree vegetation inside and on the edge of the pond
P13	8°47.05'S, 42°29.39'W	Modified passage that accumulates water surrounded by shrubs and trees
P14	8°49.34'S, 42°31.89'W	Permanent reservoir with shrub and tree vegetation inside and on the edge of the pond
P15	8°49.16'S, 42°31.65'W	Temporary pond with shrub and tree vegetation inside and on the edge of the pond

### ﻿Environmental variables of sampling points

**Figure 2.**
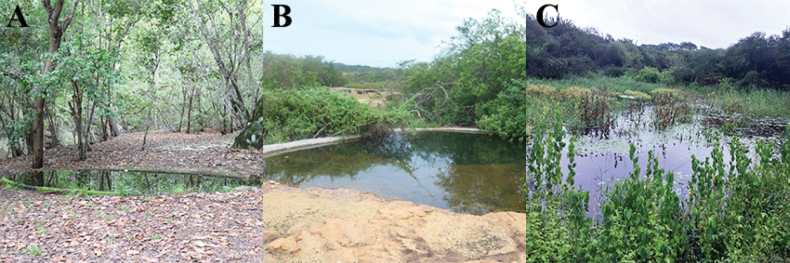
Sampled environments in the Serra da Capivara National Park, Piauí State, northeastern Brazil **A–C** represent, respectively, artificial, modified, and natural ponds.

We measured a set of nine abiotic and biotic variables at each of the 15 sampling points (Table 2). These variables were selected as they are considered strong indicators of local heterogeneity in anuran communities (e.g., Silva et al. 2011; Araújo et al. 2018; Mausberg et al. 2023). The sampling points were distributed across different habitats: three in natural ponds, three in artificial ponds, and eight in modified ponds (Fig. 2). To minimize observer bias, all variables were consistently measured by the same researcher (NLRA).

**Table 2.** List of variables recorded at each sampling point within the Serra da Capivara National Park, Piauí State, northeastern Brazil, including unit, detailed nomenclature and method.

Variable name	Definition	Unit	Method
Margin type	Pond edge characteristic	Three categories: plan (1), inclined (2) or both (3)	Visual characterization
Vegetation within the pond	Approximate percentual vegetation on pond surface	Four categories: No vegetation (0), < or = 20% (1), < or = 50% (2), > 50% (3)	Visual estimation
Types of vegetation within the pond	Characteristics of the vegetation (herbaceous, shrubby and arboreal) present in the water body	Four categories: no vegetation (0), one type of vegetation (1), two types (2), three types (3)	Visual characterization
Types of marginal vegetation	Characteristics of marginal vegetation (herbaceous, shrubby and arboreal) on the margin of the water body	Four categories: no vegetation (0), one type of vegetation (1), two types (2), three types (3)	Visual characterization
Pond localization	Characteristics of where the pond is located	Two categories: inside the cave (1), outside the cave (2)	Visual characterization
Pond number	Number of ponds present within a 200m radius of the largest pond	Three categories: one (1), two (2), more than two (3)	Visual characterization
Pond size	Surface area of the pond (m²) when full (if there is more than one pond, it will be considered the largest)	Four categories: < or = 3 m² (1), < or = 5 m² (2), < or = 10 m² (3), > 10 m² (4)	Measured using length, width and shape
Maximum pond depth	Maximum depth (m) when full (if there is more than one pond, it will be considered the largest)	Three categories: < or = 1 m (1), < or = 2 m (2), > 2 m (3)	Measured at deepest point of water body.
Pond type	Characterized based on the level of anthropic action	artificial (1), modified (2), natural (3)	Visual characterization

### ﻿Statistical analyses

We used sample-based accumulation curves (Gotelli and Colwell 2001) with 1000 randomizations based on an incidence matrix to evaluate our sampling efficiency. To estimate expected species richness in SCNP, we applied the non-parametric estimators CHAO 2 and JACKKNIFE 1 (Magurran and McGill 2011), each with 100 randomizations. To compile abundance data and avoid biases in interpretation, we used the highest abundance value recorded among the four expeditions (Andrade et al. 2019).

Considering the SCNP is a UC located in the Caatinga biome, we compared the diversity of anurans registered in the present study with 13 other localities within this biome characterized by the Caatinga sensu stricto as the predominant plant physiognomy: Picos municipality (PICOS), Piauí State (Benício et al. 2015); Seridó Ecological Station (SERID), Rio Grande do Norte State (Caldas et al. 2016); Catimbau National Park (CATNP) and Serrita municipality (SERR), Pernambuco State (Pedrosa et al. 2014; Pereira et al. 2015); São João do Cariri (SJCA) and Cabaceiras (CABAC) municipalities, Paraíba State (Vieira et al. 2009; Leite-Filho et al. 2015; Protázio et al. 2015; Cascon and Langguth 2016); Aiuaba Ecological Station (AIUAB), Rio Salgado Basin (BHRS), Middle Jaguaribe River (JAGUA), and the municipalities of Farias Brito (FBRIT) and Itapipoca (ITAP), Ceará State (Santana et al. 2015; Ávila et al. 2017; Costa et al. 2018; Castro et al. 2018; Silva-Neta et al. 2018; Oliveira et al. 2021); and Raso da Catarina Ecological Station (RCAT) and Nordestina municipality (NORD), Bahia State (Garda et al. 2013; Leite et al. 2019). For this analysis, we constructed a matrix with presence and absence data for 43 anuran species, excluding species having an uncertain specific identification (“gr.” – group, “aff.” – affinity with a known species, and “sp.” – exact species is unknown) and considering only species with an identification to be confirmed (“cf.”). Thereafter, we performed a cluster analysis by Unweighted Pair Group Average Method (UPGAM) to illustrate the similarity between the anuran composition of the SCNP and other Caatinga areas.

We first tested the normality of the variables using the SHAPIRO-WILK test and log-transformed those that did not meet normality assumptions (Shapiro-Wilk p < 0.05), which applied only to species abundance data. To detect collinearity among the variables, we calculated the Variance Inflation Factors (VIF) and excluded any variable with a VIF ≥ 10 (James et al. 2013), resulting in the removal of pond size from the analysis. We then constructed Generalized Linear Models (GLMs) to assess the effect of predictor variables – pond margin profile, percentage of vegetation within the pond, vegetation types within the pond, types of marginal vegetation, pond location, number of ponds at the sampling point, depth of the largest pond, and pond type – on response variables (anuran richness and abundance). Our general model was defined as: Response variable (richness or abundance) ~ predictor variables, family = poisson (link = “log”).

We then used Akaike’s Information Criterion with second-order bias correction for small samples (AICc) to compare models for each response variable alone or in combination (Burnham and Anderson 2002). We considered both ΔAICc and Akaike’s weight (w) of each model. Models with ΔAICc lower than 2 were interpreted as having the strongest support (Burnham and Anderson 2002). Statistical analyses were performed using the R packages vegan (Oksanen et al. 2019), bbmle (Bolker 2020), dendextend (Leonnardi et al. 2018), factoextra (Kassam et al. 2020), ggplot2 (Wickham 2016), and usdm (Naimi 2015).

## ﻿Results

**Figure 3.**
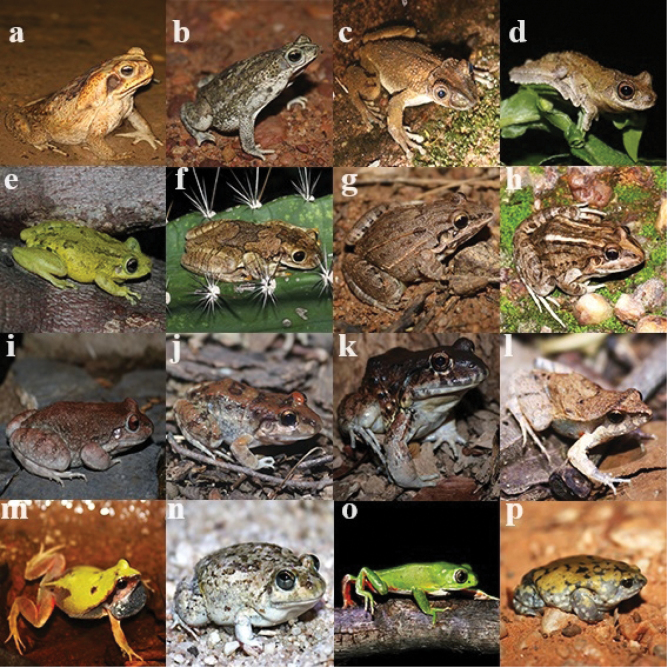
Anurans registered in the Serra da Capivara National Park, Piauí State, northeastern Brazil: **a***Rhinelladiptycha***b***Rhinellagranulosa***c***Corythomantisgreeningi***d***Dendropsophussoaresi***e***Scinaxx-signatus***f**Trachycephaluscf.nigromaculatus**g***Leptodactylusfuscus***h***Leptodactylusmacrosternum***i***Leptodactylussyphax***j***Leptodactylustroglodytes***k***Leptodactylusvastus***l***Physalaemuscicada***m***Physalaemuscuvieri***n***Pleurodemadiplolister***o***Pithecopusgonzagai***p***Dermatonotusmuelleri*.

We recorded a total of 551 individuals representing 16 anuran species across five families: Bufonidae (*N* = 2), Hylidae (*N* = 4), Leptodactylidae (*N* = 8), Microhylidae (*N* = 1), and Phyllomedusidae (*N* = 1) (Table 3, Fig. 3). The most abundant species were *Leptodactylustroglodytes* (*N* = 77), *Scinaxx-signatus* (*N* = 72), and *Pithecopusgonzagai* (*N* = 65), while Trachycephaluscf.nigromaculatus (*N* = 7) and *Pleurodemadiplolister* (*N* = 1) were the least abundant. All species are classified as “Least Concern” (LC) according to the IUCN Red List Categories and Criteria (IUCN, 2024).

**Table 3.** Anurans registered in the Serra da Capivara National Park, Piauí State, northeastern Brazil, including voucher, occurrence in the SCNP, and distribution in the Brazilian biomes.

Taxa	Voucher	Occurrence	Biome
BUFONIDAE			
*Rhinelladiptycha* (Cope, 1862)	CBPII 534	1–3, 5–11, 13–15	WD
*Rhinellagranulosa* (Spix, 1824)	CBPII 536	2, 3, 5, 6, 8–12, 14, 15	WD
HYLIDAE			
*Corythomantisgreeningi* Boulenger, 1896	CBPII 567	1, 6, 9, 10, 12	AT, CA, CE
*Dendropsophussoaresi* (Caramaschi & Jim, 1983)	CBPII 537	1–7, 9, 10	AT, CA, CE
*Scinaxx-signatus* (Spix, 1824)	CBPII 528	1–6, 9–12, 14, 15	WD
Trachycephaluscf.nigromaculatus Tschudi, 1838	CBPII 558	3	AT, CA, CE
LEPTODACTYLIDAE			
*Leptodactylusfuscus* (Schneider, 1799)	CBPII 590	10, 12, 14, 15	WD
*Leptodactylusmacrosternum* Miranda-Ribeiro, 1926	CBPII 583	10, 11, 13–15	WD
*Leptodactylussyphax* Bokermann, 1969	CBPII 569	2, 6, 8–11, 13	WD
*Leptodactylustroglodytes* Lutz, 1926	CBPII 526	1–15	WD
*Leptodactylusvastus* Lutz, 1930	CBPII 587	1, 3–11, 13–15	WD
*Physalaemuscicada* Bokermann, 1966	CBPII 585	11, 12, 15	–
*Physalaemuscuvieri* Fitzinger, 1826	CBPII 531	1, 3–9, 13, 14, 15	WD
*Pleurodemadiplolister* (Peters, 1870)	CBPII 769	11	AT, CA, CE
MICROHYLIDAE			
*Dermatonotusmuelleri* (Boettger, 1885)	CBPII 555	3, 4, 6, 7, 9, 11, 12, 15	WD
PHYLLOMEDUSIDAE			
*Pithecopusgonzagai* Andrade, Haga, Ferreira, Recco-Pimentel, Toledo & Bruschi, 2020	CBPII 548	1–6, 9–14	CA, CE

Legends: Biome distribution: Caatinga (CA), Cerrado (CE), Atlantic Forest (AT), and widely distributed (WD). See Table 1 for sampling points characterization.

**Figure 4.**
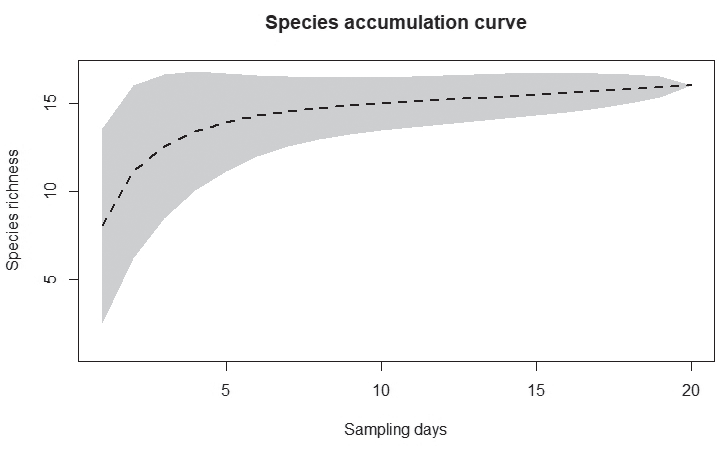
Accumulation curve for anurans sampled in the Serra da Capivara National Park, Piauí State, northeastern Brazil, based on the number of samples, constructed from 1000 randomizations.

The species accumulation curve indicated a strong tendency toward stabilization (Fig. 4), with observed species richness accounting for approximately 90% of the richness estimated by the non-parametric JACKKNIFE 1 estimator (17.9 ± 1.34) and about 95% of that estimated by CHAO 2 (16.9 ± 2.19). Consequently, we anticipate the discovery of at least two additional species in the study area.

**Figure 5.**
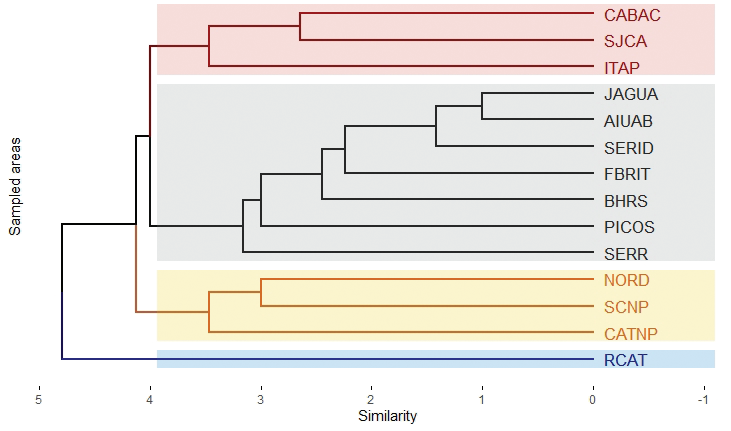
Similarity (Jaccard index and clustering method “UPGMA”) among the anuran species composition in areas of Caatinga sensu stricto.

We observed the formation of four clusters regarding the anuran composition of the Caatinga sensu stricto areas analyzed: the first one was formed by São João do Cariri and the Cabaceiras municipalities, Paraíba State, and the Itapipoca municipality, Ceará State. The second one is the largest cluster with seven areas within Ceará, Pernambuco, Piauí, and Rio Grande do Norte states. The anuran composition of the SCNP was more similar to those registered in the Catimbau National Park, Pernambuco State, and Nordestina municipality, Bahia State. The Raso da Catarina Ecological Station was isolated in the cluster analysis (Fig. 5).

Regarding the variables analyzed, we found that local habitat heterogeneity did not significantly influence anuran richness in SCNP (P > 0.05); however, it played a crucial role in explaining anuran abundance. Specifically, significant variables included margins profile (z-value = 2.907, P = 0.003), types of marginal vegetation (z-value = 2.304, P = 0.021), percentage of vegetation cover within ponds (z-value = 4.070, P < 0.001), number of ponds at the sampling point (z-value = 5.600, P < 0.001), depth of the largest pond at the sampling point (z-value = 2.991, P = 0.002), and type of ponds at the sampling point (z-value = -3.211, P = 0.001; Suppl. material 1: appendix S1). Furthermore, based on Akaike’s Information Criterion, the model incorporating all significant variables together provided a better explanation of anuran abundance in SCNP than models considering each variable in isolation (Suppl. material 1: appendix S2).

## ﻿Discussion

We identified 16 anuran species in the Serra da Capivara National Park (SCNP), which accounts for approximately 30% of the anurans known from Piauí State (Roberto et al. 2013). In addition, we recorded more than double the number of anuran species previously documented for the SCNP (Calvacanti et al. 2014). This level of species richness is considered moderate when compared to other studies conducted in areas of Caatinga sensu stricto (e.g., Pedrosa et al. 2014; Benício et al. 2015). It is interesting to highlight that the anuran composition of the SCNP was more similar to those recorded in the Catimbau National Park, Pernambuco State (Pedrosa et al. 2014) and Nordestina municipality, Bahia State (Leite et al. 2019). It is unclear why these locations are more similar, given their geographical distances. Thus, Brazilian state divisions did not seem to be predominant regarding differences in the composition of anurans in the Caatinga biome. In addition, we suggest further studies aiming to investigate the main factors filters driving the anuran composition dissimilarity in different localities of this biome.

When focusing solely on conservation units in Piauí State, the number of species in SCNP is lower than in other protected areas, such as Uruçuí–Una Ecological Station (Dal-Vechio et al. 2013; 26 species), Sete Cidades National Park (Araújo et al. 2020a; 30 species), Serra das Confusões National Park (Marques et al. 2023; 29 species), and the Environmental Protection Area Delta do Parnaíba (Araújo et al. 2020b; 33 species). Notably, except for SCNP and Uruçuí–Una Ecological Station, all other conservation units are situated within ecotonal regions of the Caatinga and Cerrado biomes. Ecotones are typically characterized by high biodiversity (Kark 2013), which may help explain the observed variation in species richness.

In terms of anuran species composition, most species identified are considered widespread across Brazilian biomes, including *Rhinelladiptycha*, *Scinaxx-signatus*, *Leptodactylusfuscus*, *L.macrosternum*, *L.syphax*, *L.vastus*, *Physalaemuscuvieri*, and *Dermatonotusmuelleri* (Frost 2024). Although we did not document any endemic species, we did encounter several species commonly associated with the Caatinga biome, such as *Corythomantisgreeningi*, *Pleurodemadiplolister*, *Pithecopusgonzagai*, *Physalaemuscicada*, and *Rhinellagranulosa* (Garda et al. 2017).

Overall, the families Leptodactylidae and Hylidae exhibited the highest diversity within the SCNP, a pattern that is frequently observed in the Neotropical region (Duellman 1978). Similar findings have been reported in the Caatinga biome (Arzabe 1999; Vieira et al. 2007; Pedrosa et al. 2014), including various conservation units in Piauí State (e.g., Araújo et al. 2020a; Marques et al. 2023). Due to the spatial segregation between leptodactylids and hylids (Protázio et al. 2015; Leite-Filho et al. 2017; Caldas et al. 2019), anurans from these families typically coexist habitually and stably in diverse environments.

We found that anuran richness in the Serra da Capivara National Park (SCNP) was not significantly influenced by local heterogeneity, regardless of whether the sampling ponds were natural, modified, or artificial. While some studies have similarly reported a lack of support for this relationship (e.g., Vasconcelos et al. 2009; Gouveia and Faria 2015), such a pattern is atypical since more heterogeneous environments generally support higher species richness (e.g., Tews et al. 2004; Andrade et al. 2016; Lorenzón et al. 2016; Araújo et al. 2018; Piña et al. 2019), particularly among anuran communities (e.g., Silva et al. 2011; Couto et al. 2017; Andrade et al. 2016, 2019; Figueiredo et al. 2019). The absence of a relationship between richness and local heterogeneity may be attributed to the prevalence of habitat-generalist species that are typical of Caatinga environments, which are present across all sampling points. These species are adapted to explore a variety of ponds within these landscapes due to their strategies for surviving in semiarid conditions. This finding aligns with Gouveia and Faria (2015), who suggested that anurans in the Caatinga exhibit stochastic usage patterns of available water bodies.

In contrast, we observed that sampling points with a higher percentage of vegetation within the ponds and a greater diversity of marginal vegetation tended to support greater anuran abundance. Additionally, the characteristics of the ponds played a significant role in influencing anuran abundance. Other studies have similarly highlighted the impact of vegetation and pond characteristics on anuran populations (e.g., Bickford et al. 2010; Dória et al. 2015; Agostini et al. 2021). Generally, more heterogeneous areas provide greater resources (MacArthur and MacArthur 1961), which can reduce both intraspecific and interspecific competition (Morin 2011). Consequently, more heterogeneous sampling areas within the SCNP facilitate the coexistence of a higher number of individuals.

This study enhances the understanding of biodiversity in the Serra da Capivara National Park by presenting an updated anuran checklist, which may inform current and future conservation strategies. Furthermore, we found that local heterogeneity influences population sizes, emphasizing the importance of heterogeneous environments in promoting stable anuran populations. Notably, artificial drinking fountains designed to support vertebrate populations during the dry season also contribute to anuran diversity, as some species utilize these structures for reproduction and establish nearby populations. Although our study is pioneering in exploring the primary drivers of anuran diversity in the SCNP, further research is essential to deepen our understanding of the ecological processes shaping these anuran communities.

## ﻿Additional information

### Author ORCIDs

Kássio de Castro Araújo https://orcid.org/0000-0003-4091-8521

Nayla Letícia Rodrigues Assunção https://orcid.org/0000-0002-6847-1269

Mirco Solé https://orcid.org/0000-0001-7881-6227

Etielle Barroso de Andrade https://orcid.org/0000-0002-5030-1675

### Data availability

All of the data that support the findings of this study are available in the main text or Supplementary Information.

## Supplementary materials

Supplementary material 1 Supplementary data Data type docx https://binary.pensoft.net/file/1321137This dataset is made available under the Open Database License (http://opendatacommons.org/licenses/odbl/1.0/). The Open Database License (ODbL) is a license agreement intended to allow users to freely share, modify, and use this Dataset while maintaining this same freedom for others, provided that the original source and author(s) are credited.Kássio de Castro Araújo, Nayla Letícia Rodrigues Assunção, Mirco Solé, Etielle Barroso de Andrade
