# Supplementary material for: ﻿Assessing the effect of local heterogeneity on anuran diversity in the Serra da Capivara National Park, Piauí State, Brazil
Source: Zookeys. 2025 May 5;1236:233–48. doi: 10.3897/zookeys.1236.138858 (PMC12070073; doi:10.3897/zookeys.1236.138858)
Supplement: Supplementary material 1 — Supplementary data [file zookeys-1236-233_article-138858__-s001.docx]

**SUPPLEMENTARY MATERIAL**

**Appendix 1**. Generalized linear models between the local environmental variables and abundance and richness of anurans in the Serra da Capivara National Park, Piauí state, Northeast Brazil

| **Abundance** AIC = 152.01 | | | | |
| --- | --- | --- | --- | --- |
| **Coefficients** | **Estimate** | **std.error** | **z-value** | **p-value** |
| (Intercept) | 0.69005 | 0.64402 | 1.071 | 0.28396 |
| Margins | 0.24538 | 0.08441 | 2.907 | 0.0365* |
| inside.vegetation | 0.67986 | 0.16704 | 4.070 | 4.70e-05* |
| inside.vegetation.type | -0.1435 | 0.12806 | -1.121 | 0.26227 |
| marginal.vegetation | 0.18014 | 0.07820 | 2.304 | 0.02124* |
| pond.location | 0.45199 | 0.34188 | 1.322 | 0.18615 |
| pond.number | 0.58286 | 0.09422 | 2.991 | 2.15e-08* |
| pond.depth | 0.28184 | 0.09422 | 2.991 | 0.00278* |
| pond.type | -0.3337 | 0.10393 | -3.211 | 0.00132* |
| **Richness** AIC = 78.984 | | | | |
| (Intercept) | 0.90170 | 1.02278 | 0.882 | 0.378 |
| Margins | 0.18306 | 0.14410 | 1.270 | 0.204 |
| inside.vegetation | 0.21845 | 0.42460 | 0.514 | 0.607 |
| inside.vegetation.type | -0.1305 | 0.31885 | -0.409 | 0.682 |
| marginal.vegetation | -0.0822 | 0.12143 | -0.677 | 0.498 |
| pond.location | 0.60614 | 0.50752 | 1.194 | 0.232 |
| pond.number | 0.14969 | 0.21167 | 0.707 | 0.479 |
| pond.depth | -0.0988 | 0.16303 | -0.607 | 0.544 |
| pond.type | -0.0591 | 0.20328 | -0.291 | 0.771 |

**Legends**: environmental variables studied were margins profile (margins), vegetation percentage within the pond (inside.vegetation), types of vegetation within pond (inside.vegetation.type), types of marginal vegetation (marginal.vegetation), localization of the pond (pond.location), number of ponds within the sampling point (pond.number), depth of the highest pond with the sampling point (pond.depth), and type of ponds with the sampling point (pond.type). Significant values (*)

**Appendix 2**. Models predicting anuran abundance according to the Akaike’s Information Criterion corrected for small samples (AICc) and Akaike’s weight (w). ΔAICc is the difference between the interest model and the model with the lowest AICc value. K represents the number of parameters, and the weights represent the relative likelihood of a model

| **Variables** | **AICc** | **ΔAICc** | **K** | **Weight** |
| --- | --- | --- | --- | --- |
| Significant.model | 167.1 | 0.0 | 7 | 1 |
| Full.model | 188.0 | 20.9 | 9 | <0.001 |
| Substrate.model | 230.2 | 63.1 | 3 | <0.001 |
| Pond.model | 232.2 | 62.5 | 4 | <0.001 |
| Vegetation.model | 258.1 | 91.1 | 4 | <0.001 |
| Margins | 258.2 | 91.1 | 2 | <0.001 |
| pond.depth | 262.2 | 95.2 | 2 | <0.001 |
| inside.vegetation | 266.9 | 99.8 | 2 | <0.001 |
| inside.vegetation.type | 273.0 | 106.0 | 2 | <0.001 |
| marginal.vegetation | 281.5 | 114.4 | 2 | <0.001 |
| pond.number | 286.8 | 119.8 | 2 | <0.001 |
| pond.location | 288.9 | 121.8 | 2 | <0.001 |
| pond.type | 301.6 | 134.6 | 2 | <0.001 |
| Null.model | 306.1 | 139.1 | 1 | <0.001 |

**Legends**: Model with significant variables obtained in the GLM (Significant.model), full model considering all eight variables (Full.model); model with margins profile and pond location (Substrate.model); model with pond characteristics: number of ponds, pond size and depth (Pond.model), model with vegetational structure: vegetation percentage within the pond, types of vegetation within pond, and types of marginal vegetation (Vegetation.model); and null model (Null.model). Models with each variable isolated were named according to Appendix 1.
